# International HIV Dementia Scale for HIV-Associated Neurocognitive Disorders: A Systematic Review and Meta-Analysis

**DOI:** 10.3390/diagnostics11061124

**Published:** 2021-06-20

**Authors:** Elena Cecilia Rosca, Philippe Tadger, Amalia Cornea, Raluca Tudor, Cristian Oancea, Mihaela Simu

**Affiliations:** 1 Department of Neurology, Victor Babes University of Medicine and Pharmacy of Timisoara, 300041 Timisoara, Romania; amalia.cornea@yahoo.com (A.C.); tudor.raluca@yahoo.com (R.T.); mihaelasimu6713@gmail.com (M.S.); 2 Department of Neurology, Clinical Emergency County Hospital Timisoara, 300736 Timisoara, Romania; 3 Neuroscience Research Center Timisoara, Clinical Emergency County Hospital Timisoara, 300736 Timisoara, Romania; 4 Independent Researcher, Belgium; philippetadger@gmail.com; 5 Center for Research and Innovation in Precision Medicine of Respiratory Diseases, University of Medicine and Pharmacy “Victor Babes” Timisoara, 300173 Timisoara, Romania; oancea@umft.ro

**Keywords:** International HIV Dementia Scale, HIV-associated neurocognitive disorder, systematic review, meta-analysis

## Abstract

The present study aims to systematically review the evidence on the accuracy of the International HIV Dementia Scale (IHDS) test for diagnosing human immunodeficiency virus (HIV)-associated neurocognitive disorders (HAND) and outline the quality and quantity of research evidence available on the accuracy of IHDS in people living with HIV. We conducted a systematic literature review, searching five databases from inception until July 2020. We extracted dichotomized positive and negative test results at various thresholds and calculated the sensitivity and specificity of IHDS. Quality assessment was performed according to the Quality Assessment of Diagnostic Accuracy Studies 2 (QUADAS-2) criteria. Fifteen cross-sectional studies, published between 2011 and 2018, met the inclusion criteria for meta-analysis. Overall, 3760 patients were included, but most studies recruited small samples. We assessed most studies as being applicable to the review question, though we had concerns about the selection of participants in three studies. The accuracy of IHDS was investigated at thirteen cut-off points (scores 6–12). The threshold of 10 is the most useful for optimal HAND screening (including asymptomatic neurocognitive disorder, symptomatic HAND, and HIV-associated dementia) with fair diagnostic accuracy.

## 1. Introduction

Despite the recent advances in the immunovirological management of individuals with human immunodeficiency virus (HIV) infection, HIV-associated neurocognitive disorders (HAND) in adults are estimated to occur in between 30% and 60% of individuals [1,2,3,4,5,6]. Epidemiological studies reported that HIV-associated dementia (HAD) is rare (2–4%) [6], most patients presenting milder forms of HAND, including asymptomatic neurocognitive impairment (ANI) and mild neurocognitive disorder (MND) [3,7,8]. A recent systematic review found that the global prevalence of HAND was 42.6%; the milder forms of cognitive impairment, including ANI and MND, accounted for approximately 88% of all HAND forms, while the most severe form, HAD, was rare [9]. The prevalence of HAND and ANI in people living with HIV (PLWH) was higher in Latin America and the Caribbean and among individuals with a low level of nadir CD4 count (<200 cells/mm^3^). The prevalence of total HAND did not differ by the proportion of participants receiving antiretroviral treatment (ART), current CD4 count, or proportion of the participants with HCV co-infection. Prevalence estimates for specific HAND subtypes were 23.5% for ANI, 13.3% for MND, and 5% for HAD. The number of MND and HAD cases decreased with the level of income, current CD4 count, and proportion of ART. The prevalence of ANI increased with age, whereas the prevalence of MND and HAD decreased with age [9].

Before the introduction of combination ART, many patients developed severe neurological impairment in the final months of their illness, comprising cognitive, behavioral, and motor symptoms. The cognitive impairment consisted mainly of mental slowing and attention and memory dysfunction. Since the introduction of ART, the incidence of dementia has decreased. Today, patients with treatment and long-term infection present milder cognitive symptoms. In addition, a shift has occurred in certain demographic variables and risk factors, such as increased age and cardiovascular risk factors [10]. Therefore, the differential diagnosis of the cognitive dysfunction of HIV-infected patients needs to include virus-independent and age-associated diseases [11]. Thereby, the neuropsychologic profile of HAND has broadened [12,13]. Patients present a subcortical profile of cognitive impairment, the core deficits consisting of mental slowness, attention and memory dysfunction, and impaired executive functions [13]. One of the most frequent cognitive abnormalities in HAND consists of decreased information processing speed [12,13,14]. Because mental speed facilitates most cognitive and motor processes, some authors even consider it the key deficit, which leads to impairments in other cognitive domains [15]. In addition, patients with HAND present impaired attention and working memory, which are closely related and co-occur [13,14]. They have deficits in learning new information and prospective episodic memory, with impaired ability to execute a future intention or “remembering to remember” [14]. In addition, patients with HAND might present executive dysfunction, with deficits in reasoning, planning, problem-solving, and shifting between tasks [14,16]. In the language domain, the most frequent finding is impaired fluency, although this could also be due to mental slowness or executive dysfunction [14,16]. More rarely, patients with HAND may present sensory–perceptual impairments, with disturbances in interpretation and integration of auditory, visual, or sensorial stimuli [14].

Currently, HAND is classified according to the Frascati criteria, with three different degrees of cognitive impairment that are separately diagnosed. In patients with ANI, the neuropsychological test performance is one standard deviation (SD) below the normative data in at least two of five cognitive areas, with intact daily functioning. The MND is characterized by similar neuropsychological test results, with impaired daily functioning. HAD is characterized by severe deficits in at least two cognitive domains, typically two SDs below normative data, and more severe daily functioning impairment [17].

Recently, the validity of the Frascati criteria has been challenged [18,19,20]. Researchers disagree over the clinical relevance of ANI and the validity of neuropsychological testing in characterizing the cognitive deficits. They argue against testing for ANI since there are no screening tools with high sensitivity and specificity that can be utilized in all clinical settings. There is no consensus on the therapeutic management of asymptomatic patients. Furthermore, screening can lead to unnecessary and expensive diagnostic procedures, and a positive result might cause distress to some people living with HIV [19]. In addition, longitudinal studies only rarely documented the progression of ANI to a symptomatic status [21], and some observational studies did not find an association between combination ART with the estimated high central nervous system (CNS) effectiveness and neurocognitive function [1,22,23].

On the other hand, some arguments support screening for ANI. Several studies have demonstrated that patients with ANI have poor medication adherence and high unemployment rates [24]. In addition, ANI might be associated with an increased risk of progressive neurocognitive disease [21]. Some studies have reported that ART with high CNS effectiveness is associated with improvement in cognitive function [25]; changing ART based on estimated CNS effectiveness determined a decline of the levels of HIV RNA in the cerebrospinal fluid (CSF), with the improvement of cognitive functions [26]. Furthermore, some ART was demonstrated to be neurotoxic [27]. Although it has been argued that, since ANI is “asymptomatic”, it may have little clinical significance, recent research reported in patients with ANI the presence of grey and white matter abnormalities [28], along with abnormal blood plasma biomarkers (e.g., nadir CD4 count, neopterin, neurofilament light chains) [5].

In general, the international guidelines agree on the recommendations regarding HAND diagnosis (for a review, see Underwood & Winston 2016) [29]. Some guidelines have a specific section regarding the diagnosis and management of cognitive impairment; they recommend a comprehensive assessment including a thorough medical history and examination, screening for depression, neuropsychological testing, cerebral magnetic resonance imaging (MRI), and lumbar puncture [30,31,32]. Nonetheless, there is no clear consensus on the specific tests that should be used as part of the neuropsychological assessment. All guidelines refer to the Frascati criteria, which recommend a complex neuropsychological assessment, testing several cognitive domains, with the endorsement of several preferred tests for each cognitive domain [17]. In addition, the Mind Exchange Working Group advises that the tests should be validated in the language and culture of the population and scored according to appropriate normative data [31].

Notwithstanding, such tests are not available in many centers, and their use requires highly trained personnel [17]. Therefore, brief cognitive screening instruments that are sensitive, easily accessible, and can be administered by healthcare professionals across a range of settings would be useful. Nonetheless, most HIV guidelines do not make any specific recommendations on screening for neurocognitive impairment. There are considerable differences among the guidelines that propose recommendations, reflecting the uncertainties in the literature [29]. The European AIDS Clinical Society (EACS) guidelines (v.10.1, EACS 2020) recommend screening all HIV-positive individuals without highly confounding conditions (such as severe psychiatric diseases, abuse of alcohol or psychotropic drugs, current CNS opportunistic infections or other neurological diseases, sequels of CNS disorders) at HIV diagnosis, before ART initiation, and then later as indicated based on symptoms. The EACS screening method consists of asking three questions: “Do you experience frequent memory loss?”, “Do you feel that you are slower when reasoning, planning activities, or solving problems?”, and “Do you have difficulties paying attention?”. If a patient answers “Yes” to at least one of these questions, the screening test is considered positive, and further assessment is recommended [30].

However, the consensus report of the Mind Exchange Program recommends screening within six months of diagnosis, before ART initiation, every 6–12 months if there is a high risk, every 12–24 months if there is low risk, and immediately if there is any clinical deterioration [31]. The screening test selection depends on the availability of a clinician suitably trained to administer and interpret each instrument and whether the physician intends to screen for HAD or the milder forms of HAND. In addition, other considerations include financial costs, time, and the characteristics of the population in which the tool is intended for use. Nevertheless, the neuropsychological resources are limited in many settings worldwide; therefore, a probable clinical diagnosis of HAND can be based on symptom questionnaires, functional assessments, screening tools, or a limited neuropsychological assessment. Patients with particular features could then be referred for an extensive neuropsychological assessment [31]. In addition, the Mind Exchange Working Group specifies some preferred screening tests, such as the HIV Dementia Scale (HDS) and the International HIV Dementia Scale (IHDS).

The British HIV Association (BHIVA) recommends that HIV-positive individuals should be screened for cognitive impairment within the first three months of receiving the diagnosis of HIV infection. Furthermore, all HIV-positive patients should be screened following events known to trigger or exacerbate cognitive dysfunctions and otherwise on an annual basis [33]. These recommendations are similar to those of the Infectious Diseases Society of America guidelines [34]. The World Health Organization (WHO) endorses routine screening for people from key populations living with HIV to optimize health outcomes and improve adherence to ART. Nonetheless, the screening method and frequency are not specified [35]. The Italian Society for Infectious and Tropical Diseases recommends screening all PLWH with cognitive complaints. Among the suggested tests, they recommend the Cogstate and Montreal Cognitive Assessment (MoCA) [32].

The guidelines also endorse a neurological examination, cerebral MRI, and CSF examination in order to exclude other pathologies if the neuropsychological impairment detected on screening is confirmed by tests exploring multiple cognitive domains. In addition, an assessment of CSF HIV viral load level is recommended, and, where appropriate, evidence for genotypic drug resistance (GDR) in a paired CSF and plasma sample should be performed [30]. After additional causes of cognitive dysfunction are excluded, and HAND is diagnosed, the clinician must take specific treatment and care measures [30].

To date, only a few screening tools have been developed and validated, including the HDS and its derivative form, IHDS [36,37]. Both instruments are relatively insensitive to the milder cognitive symptoms that predominate in the combination ART era [38,39]. Although they are recommended as screening tools by expert HIV guidelines [31], recent systematic reviews concluded that their accuracy is low [38,39]. Summary estimates for the HDS as a test for HAND presented sensitivity and specificity of 42% and 91%, respectively [38]. Another meta-analysis found similar results: the HDS presented a poor pooled sensitivity of 48% [39].

The IHDS was designed for use in international, resource-limited settings as a screening tool under different cultural, linguistic, and educational conditions. The scale evaluates memory, motor speed, and psychomotor functioning [36]. It can be easily incorporated within a clinical visit and does not require specific training.

The IHDS consists of three subtests: timed finger tapping, timed alternating hand sequence test, and recall of four items at 2 min [36]. On the timed finger tapping subtest, the patient is asked to open and close the first two fingers of the non-dominant hand as widely and as quickly as possible over a 5 s period. The maximum score is 4 points (i.e., 15 finger taps/5 s). The second subtest consists of the assessment of psychomotor speed. The patient is instructed to perform the following movements with the non-dominant hand as fast as possible over a 10 s period: (i) clench the hand in a fist on a flat surface; (ii) put the hand flat on the surface with the palm down; and (iii) put the hand perpendicular to the flat surface on the side of the fifth digit. The three hand positions are initially demonstrated to the participant by the examiner, and then the participant should perform the sequence correctly twice for practice before the 10 s subtest is performed. The maximum score on this task is 4; it is attributed if the patient correctly performs four sequences within 10 s. The third subtest of IHDS consists of the assessment of verbal recall. Registration (new learning) is measured by reciting four words to the patient and then asking him to repeat them immediately. The examiner should repeat the words until the subject correctly repeats all four words. Then, the patient is asked to recall the four words after the timed finger tapping, and alternating hand sequence tests are performed. The number of items recalled is scored out of 4. If the subject does not recall the words, he is prompted with a semantic clue. A half-point is assigned for each correct word recalled after prompting. The maximum score is 12 [36].

The IHDS was found to have a pooled sensitivity of 62% [39]. For detecting HAD, the scale presented a sensitivity of 74.3% and a specificity of 54.7%. The sensitivity and specificity for MND were 64.3% and 66.0% [38].

Other general cognitive screening tests, such as Mini-Mental State Examination (MMSE) and MoCA, have been used in clinical practice in various neurological disorders. Although MMSE is widely used as a screening tool for HAND, studies have indicated that it is not very reliable in detecting cognitive impairment in PLWH [40,41,42,43]. The MoCA has been used in patients with HIV as a screening instrument with variable results. A recent systematic review found that a lower threshold than the original cut-off of 26 is probably more useful for HAND screening; the optimal cut-off score that offered the best balance between true-positive and false-positive results was reported to be 23 [44].

An early diagnosis that enables specific treatment and care of HAND is essential. Whereas all guidelines recommend for HAND diagnosis the Frascati criteria, with extensive neuropsychological testing, this is time-consuming, expensive, necessitates trained personnel, and it is not available in many centers. Therefore, screening for cognitive impairment would help to identify the patients that should be further investigated. Nonetheless, the available guidelines on screening for HAND reflect the uncertainties in the literature, and clinicians are faced with a difficult choice: which screening test should they use. IHDS fulfills important feasibility criteria for use in clinical practice: it does not require knowledge of the English language, it has a short administration time (2–3 min), it can be easily performed by non-neurologists in an outpatient setting, and it requires no special instrumentation other than a watch with a second hand. Therefore, it is ideal for an international setting where resources may be limited [36]. In addition, the IHDS assesses cognitive domains that were demonstrated to be frequently impaired in HAND.

Although several studies have explored the utility of the IHDS to detect cognitive impairment, the sensitivity and specificity values and the cut-off scores have differed across studies. Even though the diagnostic assessment pathways may vary across different countries, usually HAND is screened in specialized infectious diseases clinics during outpatient visits. The IHDS could be a helpful screening instrument in identifying individuals with cognitive impairment that require further assessments and specific care, facilitating access to appropriate services. Notwithstanding, false-positive results imply high costs and harm due to further unnecessary investigations and psychological distress. Therefore, there is considerable value in determining the strength of the evidence that supports the use of IHDS as a screening test for HAND. We aim to systematize evidence from different studies, integrate the existing information, and provide data for rational decision-making, highlighting possible answers accessible to clinicians and health care providers.

This systematic review aims to evaluate research regarding the accuracy of the IHDS against a concurrently applied reference standard and to highlight the quality and quantity of evidence available in this regard. Additionally, we aim to identify the gaps in the literature regarding this short screening test.

## 2. Materials and Methods

The present systematic review and meta-analysis were performed following the recommendations described in the Cochrane Handbook for Diagnostic Test Accuracy Reviews [45]. Results were reported according to the guidelines of the Preferred Reporting Items for Systematic Reviews and Meta-Analysis [46].

The protocol was registered to PROSPERO (protocol number CRD42019131113).

### 2.1. Search Strategy and Selection Criteria

A computerized bibliographic search was performed from inception to July 2020 on the following databases: MEDLINE/PubMed, Scopus, Cochrane Library, Latin American and Caribbean Health Sciences Literature (LILACS), and PsychINFO. In addition, we also checked reference lists of all relevant research papers in order to identify possible additional studies.

The following keywords were used: “International HIV Dementia Scale” OR the acronym “IHDS,” AND “HIV infection” [MeSH] and “acquired immunodeficiency syndrome” [MeSH]. These search terms were for PubMed, the primary source of citations. Searches in other data sources used similar versions of these terms, appropriate for each database. We did not apply search filters (collection of terms aimed at reducing the number of papers needed to be screened), because our aim was to generate a comprehensive list of studies that would be suitable for answering the research question. Even the most sensitive filters have been reported to miss relevant studies and perform inconsistently across subject areas and study designs. At the same time, they have not significantly reduced the number of studies that need to be assessed [45,47]. In addition, we did not apply any language restrictions to our search.

Two authors reviewed the title, abstract, and full text (when needed) of all retrieved papers and determined whether the study met the inclusion criteria. During the abstract review stage, in order not to miss any potentially eligible studies, we did not exclude the papers if we were not sure whether there was an appropriate reference standard or index test and if we were uncertain if the article was a diagnostic test accuracy study. We evaluated all these papers in full text. The participation of a third rater was used to address discrepancies.

Eligible studies were cross-sectional studies in which participants received the index test and the reference standard diagnostic assessment. Case-control studies were excluded owing to a high possibility of bias. We included studies reporting adults (over 18 years old) with confirmed HIV infection in which the association between IHDS score and HAND was assessed, IHDS being used as an index test. Although we expected to find the recommended cut-off score of 10 or below to differentiate normal (11 and above) from impaired cognition, we also included studies using other thresholds (6–12). The target condition was HAND, including ANI, MND, and HAD, as classified by the Frascati criteria [17]. We used as a reference standard for HAND a complex neuropsychological assessment, evaluating at least five neurocognitive domains (including verbal and language, attention and working memory, abstraction and executive function, learning and recall, speed of information processing, and motor skills), with consensual recommendations on appropriate tests. As recommended by international guidelines, neurocognitive impairment was defined as an impairment in cognitive function on the above neuropsychological tests in which performance is considered clinically significant compared to appropriate controls matched by age and educational level [17,30,31]. We excluded studies of participants with confounding factors such as neurological disorders (e.g., recent traumatic brain injury, CNS infections, stroke, neurodegenerative disorders, and brain tumors), active psychosis, significant substance abuse, including alcohol and recreational drugs, and active infections.

Disagreements were resolved through discussion. The methodological quality of the studies was assessed by two authors independently, according to the Cochrane Collaboration’s tool for assessing the risk of bias [45]. We used the unmodified Quality Assessment of Diagnostic Accuracy Studies 2 (QUADAS-2) tool [48].

### 2.2. Data Extraction and Statistical Analysis

Two reviewers independently extracted data from individual studies into two-by-two tables. A third reviewer resolved any discrepancies. We calculated for each cut-off score the following parameters: sensitivity (proportion of individuals diagnosed with cognitive impairment who tested positive on the IHDS), specificity (proportion of patients indicated as normal, who tested negative on the IHDS), the positive predictive value (PPV —the proportion of individuals with a positive IHDS test who were diagnosed with cognitive impairment), and negative predictive value (NPV—the proportion of patients who tested negative on the IHDS, without cognitive disturbances). In addition, we calculated the likelihood ratios for positive results (LR+), representing the probability that a patient diagnosed with cognitive impairment could test positively on the IHDS as well as the likelihood ratios for negative results (LR−), referring to the probability that an individual diagnosed with cognitive dysfunction could present a negative IHDS result.

Further calculations included the Youden index, an estimated value of the optimal threshold at which sensitivity (true positive patients) is maximized and false-positive results are minimized. Youden’s index evaluates the overall discriminative power of a diagnostic procedure. It is calculated by deducting one point from the sum of a test’s sensitivity and specificity; the index is expressed not as a percentage, but as a part of a whole number: (sensitivity + specificity)—1. Youden’s index equals 0 in a test with poor diagnostic accuracy, and in a perfect test, Youden’s index equals 1 [49].

A bivariate multi-level (multi-cut-off) random-effects model was used to model to fit the multiple thresholds data of the primary studies; such models were fitted using the “diagmeta” package in R [50]. The response variables are the false-negative rate and the true-negative rate, which are modeled using the study as the grouping factor and allowing data from multiple thresholds for each study. Each model can have a random intercept and a random slope (or not) which can be different or similar for each response variable. The n-random effects are assumed to have an n-dimensional multivariate normal distribution; these are used to generate an SROC curve and pool estimation in each threshold and the optimal threshold.

The “diagmeta” program offers 16 different possible models; half with the assumption of equal variances of the diagnostic tests in both groups, and the others not; the eight available basic mixed linear models (“DIDS”, “CIDS”, “DICS”, “CICS”, “DS”, “CS”, “DI”, and “CI”) are derived from different assumptions about common/different random intercept/slope for each response variable. After successful convergence, the AIC criterion was used for model selection in each diagnostic subgroup. The advantage of the multi-cutoff model is that it uses all the available information reported on each threshold explicitly, avoiding the bias and overestimation that may occur selecting a unique threshold per study. Furthermore, using the information of each study enables the estimation of the optimal cut-off for the index tests. The multi-cut-off model is based on a parametric assumption for the diagnostic test (normal or logistic), which is very common [50]. Additional data on model selection can be found in the Supplementary Materials (Tables S1–S3).

Additionally, a non-parametric random effect model was implemented [51] using the package nsROC::metaROC. The advantage of the multi-cutoff model over the non-parametric is that the former can provide us an optimal cut-point. The advantage of the latter is to give us a closer fit to the observed data so that it can be used as a point of comparison concerning the estimates (AUC, sensitivity, and specificity).

## 3. Results

### 3.1. Included Studies

From a total of 54 unique studies identified using the search strategy and assessed in full-text, we included in our systematic review and meta-analysis 15 studies [52,53,54,55,56,57,58,59,60,61,62,63,64,65,66]. The characteristics of the studies are summarized in Table 1.

**Figure 1:**
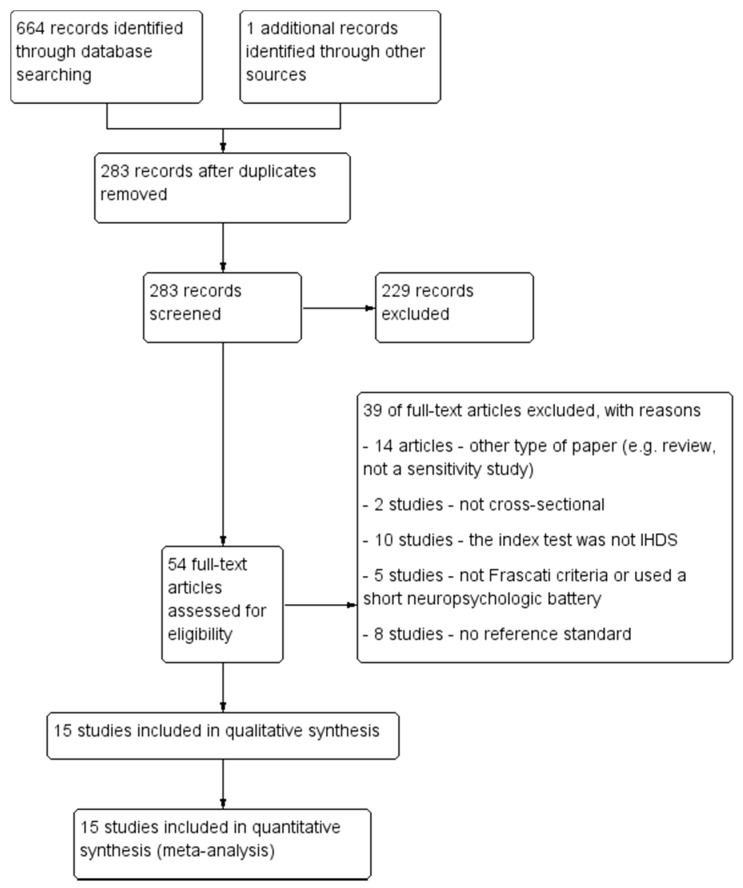
The PRISMA selection flow chart.

The PRISMA diagram describing the selection process of studies is detailed in Figure 1.

Thirty-nine studies were excluded for the following reasons: the study did not present a cross-sectional design (*n* = 2), inadequate reference standard (*n* = 5) or absence of a reference standard (*n* = 8), the IHDS was not the index test (*n* = 10), or the research paper was not a diagnostic test accuracy study (*n* = 14).

### 3.2. Methodological Quality of Included Studies

**Figure 2:**
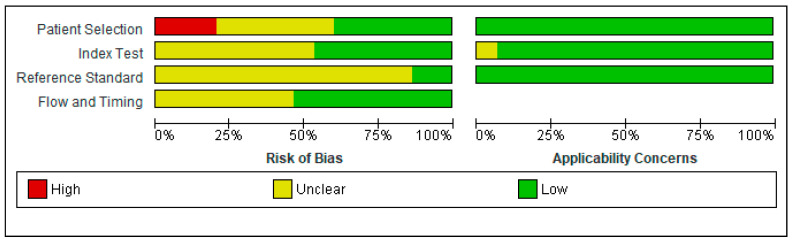
Risk of bias and applicability concerns graph: review authors′ judgments about each domain presented as percentages across included studies.

**Figure 3:**
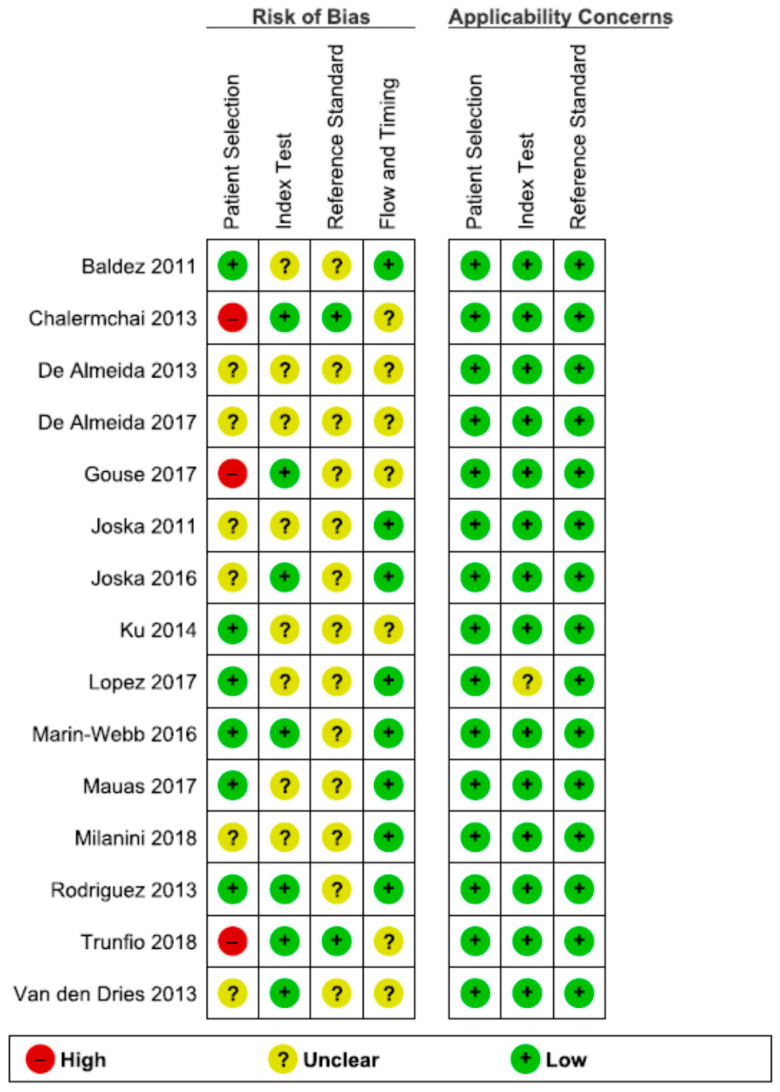
Risk of bias and applicability concerns summary: review authors′ judgments about each domain for each included study.

The QUADAS-2 scores for each domain are presented in Figure 2 and Figure 3.

In the Patient Selection domain, the risk of bias was reduced by selecting only cross-sectional studies. A random or consecutive sample of patients was reported in eight studies [53,56,57,59,60,63,64,66]. One study enrolled the patients based on CD4 count (<350 cells/mm^3^) [61]; one study included only PLWH with moderate to severe HAND [55]. One study enrolled only subjects presenting with an altered IHDS score (≤10) or complaining of neurocognitive symptoms [53]. Therefore, these studied were considered to present a high risk of bias, investigating a highly selected population.

All patients were recruited in outpatient clinics from urban areas.

Regarding the Index Test domain, eight studies were considered to be presenting an unclear risk of bias [52,54,56,57,60,62,65,66]. In eight studies, it was unclear if the index test results were interpreted without knowledge of the results of the reference standard [52,54,56,57,60,62,65,66]. Five studies specified the order of the neuropsychological tests. Therefore, the IHDS must have been interpreted without knowledge of the extensive neuropsychological battery results [55,58,59,63,64].

Regarding the Reference Standard domain, all studies used a reference standard that would correctly diagnose HAND. However, only two studies specified that the reference standard was interpreted without knowledge of the index test results [53,61]. Therefore, all the other studies were classified as having an unclear risk of bias.

The flow and timing of the cognitive tests were unclear in seven studies [53,54,55,60,61,62,64].

There were no exclusions from the analysis in twelve studies [52,53,54,55,57,58,59,61,62,63,65,66].

Generally, the studies had a low risk of bias, and no study had more than one QUADAS-2 item assessed as having a high risk of bias.

### 3.3. Findings

In general, 15 studies that assessed 3760 patients were included. There was an overlap of participants because of the use of patients across several studies where multiple cut-off points were examined. The publication year ranged between 2011 and 2018. The study samples were selected from five continents: Europe (Italy, Germany, Netherlands), North America (USA), South America (Brazil, Argentina), Africa (East Africa, South Africa), and Asia (Korea, Thailand). Samples ranged in size (from 45 to 2208 participants) and gender (12.8% males to 98.8% males), median age (29.75 to 53 years), educational level, CD4 values, and viral load. All patients were on ART only in three studies [56,58,64]. Two studies included only ART naïve patients [61,65]. All the studies used as reference standard the Frascati criteria, with extensive neuropsychological batteries (ranging from 6 to 27 cognitive tests) measuring multiple cognitive domains (ranging from 6 to 10). The characteristics of the included studies are presented in Table 1.

We present a separate analysis for each type of diagnosis in the following subsections: HAND, symptomatic HAND, and HAD. We have performed a subgroup analysis only for HAND diagnosis because there was a limited number of studies for the other types of diagnosis (symptomatic HAND and HAD). There are no standardized methods or tests for the multi-cutoff models to evaluate the heterogeneity (or their source) or the outliers formally; therefore, only subjective methods are presented.

#### 3.3.1. IHDS for Detecting HAND

Data on the accuracy of IHDS for detecting HAND were provided in 13 studies [52,53,54,56,57,58,59,60,62,63,64,65,66].

The accuracy of IHDS at different thresholds, from 6 to 12, is provided in Table 2.

**Figure 4:**
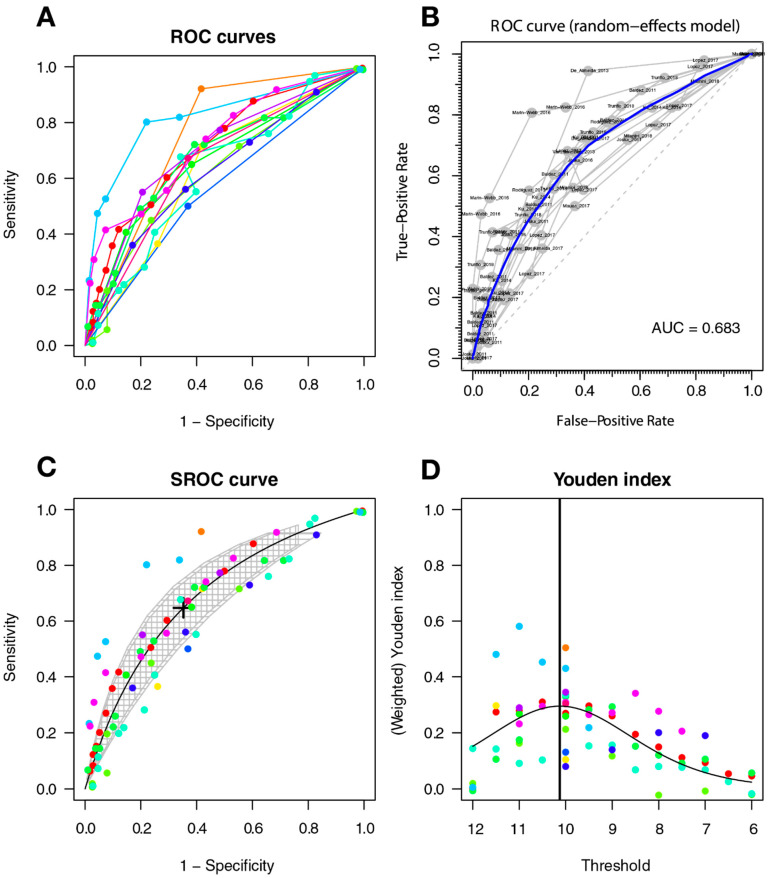
IHDS for screening HAND. Individual studies are displayed as colored lines. Each point represents a reported threshold value; points of the same color represent thresholds reported within the same study. The cross in the SROC curve indicates the Youden-based threshold value: sensitivity of 0.647 (95%CI 0.541–0.741), specificity of 0.647 (95%CI 0.522–0.755), and the AUC = 0.693 (with a CI: [0.626–0.754] [0.614–0.763]). (**A**). Descriptive ROC curve. (**B**). ROC—random-effects model. The points are the pairs of the true-positive and false-positive rates used in the meta-analysis. (**C**). SROC curve with estimation and confidence interval as grey area. (**D**). The estimation of the Youden index.

The optimal threshold for screening for HAND was 10.127, with a sensitivity of 0.647 (95%CI 0.541–0.741), a specificity of 0.647 (95%CI 0.522–0.755), and an AUC of 0.693 (with a CI: [0.626–0.754] [0.614–0.763]); the AUC for the non-parametric ROC model is 0.683, which contains a multi-cutoff model confidence interval (Figure 4).

#### 3.3.2. IHDS for Detecting Symptomatic HAND

The present systematic review found four studies investigating the use of IHDS for detecting symptomatic HAND, including overall 699 patients with HIV [53,58,61,66].

The estimated accuracy of IHDS at different thresholds, from 6 to 12, is provided in Table 3.

**Figure 5:**
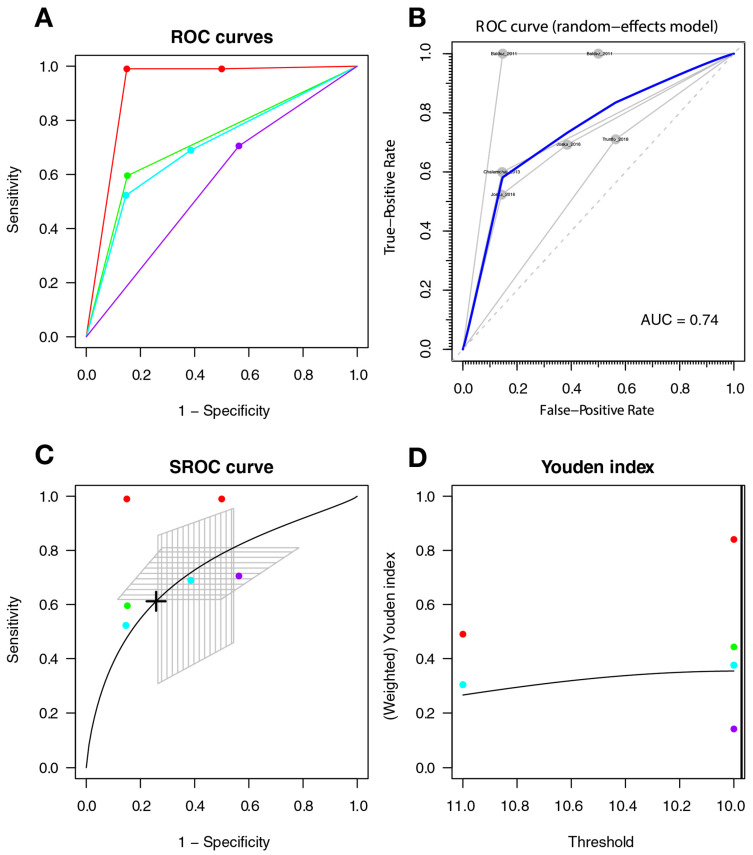
The IHDS for screening symptomatic HAND. Individual studies are displayed as colored lines. Each point represents a reported threshold value; points of the same color represent thresholds reported within the same study. The cross in the SROC curve indicates the Youden-based threshold value: sensitivity = 0.612 (95%CI 0.299–0.854), specificity = 0.742 (95%CI 0.509–0.889), and AUC = 0.725 (with a CI: [0.327–0.931] [0.512–0.83]) (**A**). Descriptive ROC curve. (**B**). ROC—random-effects model. The points are the pairs of the true-positive and false-positive rates used in the meta-analysis. (**C**). SROC curve with estimation and confidence interval as grey area. (**D**). The estimation of the Youden index.

The optimal cut-off point was 9.971, with a sensitivity of 0.612 (95%CI 0.299–0.854), a specificity of 0.742 (95%CI 0.509–0.889), and AUC = 0.725 (with a CI: [0.327–0.931] [0.512–0.83]); the AUC for the non-parametric ROC model was 0.793 (Figure 5).

#### 3.3.3. IHDS for Detecting HAD

Six studies provided data on the use of IHDS in detecting HAD [52,55,58,59,63,66].

The estimated specificity and sensitivity of IHDS at different thresholds are presented in Table 4.

**Figure 6:**
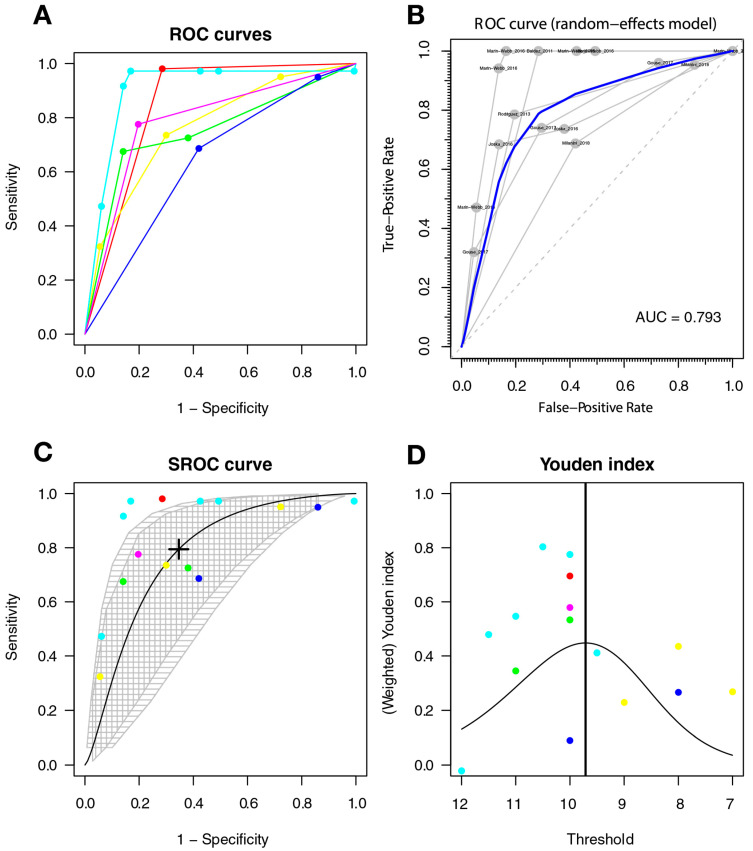
The IHDS for screening HAD. Individual studies are displayed as colored lines. Each point represents a reported threshold value, points of the same color represent thresholds reported within the same study. The cross in the SROC curve indicates the Youden-based threshold value: sensitivity = 0.794 (95%CI 0.468–0.944], specificity = 0.654 (95%CI 0.335–0.876), and AUC = 0.777 (with a CI: [ 0.597–0.893] [0.554–0.91]). (**A**). Descriptive ROC curve. (**B**). ROC—random-effects model. The points are the pairs of the true-positive and false-positive rates used in the meta-analysis. (**C**). SROC curve with estimation and confidence interval. as grey area. (**D**). The estimation of the Youden index.

The optimal cut-off point was 9.72, with a sensitivity of 0.794 (95%CI 0.468–0.944], a specificity of 0.654 (95%CI 0.335–0.876), and an AUC = 0.777 (with a CI: [0.597–0.893] [0.554–0.91]); the AUC for the non-parametric ROC model is 0.793, which is contained multi-cutoff model confidence interval (Figure 6).

#### 3.3.4. Overall Accuracy of IHDS

In accordance with the pooled estimation of the accuracy of IHDS for detecting the cognitive impairment in different groups, including HAND, symptomatic HAND, and HAD, we propose a cut-off score of 10 as a common threshold for all the groups. Assuming a score of 10 as the common threshold, the accuracy measures would be:

- HAND: true positive rate (TPR) = 0.619 (95% CI 0.511–0.717), true negative rate (TNR) = 0.675 (95% CI 0.552–0.777), with a sensibility of 0.619 (95% CI 0.551–0.717), and a specificity of 0.675 (95% CI 0.552–0.777).
- Symptomatic HAND: TPR = 0.618 (95%CI 0.308–0.855), TNR = 0.736 (95%CI 0.503–0.884), with a sensibility of 0.618 (95%CI 0.308–0.855), and a specificity of 0.736 (95%CI 0.503–0.884).
- HAD: TPR = 0.856 (95% CI 0.570–0.964), TNR = 0.581 (95% CI 0.270–0.838), with a specificity of 0.856 (95% CI 0.570–0.964), and a sensibility of 0.581 (95%CI 0.270–0.838).

#### 3.3.5. Heterogeneity of Studies

The ROC curves show subjectively greater heterogeneity for symptomatic HAND and HAD, with greater variability between the ROC lines of each study; there is less heterogeneity between the studies included in the HAND model (see Figure 4, Figure 5 and Figure 6). An additional sign of heterogeneity is the difference between the estimated and predicted CI of each SROC model, which also confirms a greater heterogeneity in the group of symptomatic HAND, and HAD, and finally HAND with the lowest one. Two studies can be assumed as outliers for HAND [59,62] and two as outliers for the HAD model [59,66]. Finally, the study of Baldez [66] can be considered an outlier for symptomatic HAND.

#### 3.3.6. Sensitivity Analysis

We conducted a sensitivity analysis to examine the impact of different subgroups on the meta-analytic findings. However, the sensitivity analysis was possible only for HAND; for the other types of diagnosis (i.e., symptomatic HAND and HAD), we had only a limited number of available studies.

The variables considered for subgroups were

•The risk of bias

We performed a sensitivity analysis excluding the studies found with a high risk of bias on QUADAS-2 [53,55,61] (see Table 5 and Supplementary Figure S1).

•The Frascati criteria (<7 cognitive domains vs. ≥7 cognitive domains)

We investigated whether the number of cognitive domains investigated by the reference standard assessment (Frascati criteria) impacted the optimal threshold (Table 6, Supplementary Figure S2).

•The number of cognitive tests (≤10 tests vs. >10 tests)

We performed subgroup analysis, grouping the studies depending on the number of conative tests that authors used as a reference standard (Table 7, Supplementary Figure S3).

•The number of CD4 (>500 vs. <500)

We analyzed whether the number of CD4 cells impacts the psychometric properties of the IHDS (Table 8).

•ART therapy (≤75% of patients on ART vs. >75% of patients on ART)

We investigated if the use of ART has an impact on the IHDS scores (Table 9).

The SROC models for each subgroup are presented in the Supplementary Material (Supplementary Figures S4 and S5).

**Figure 7:**
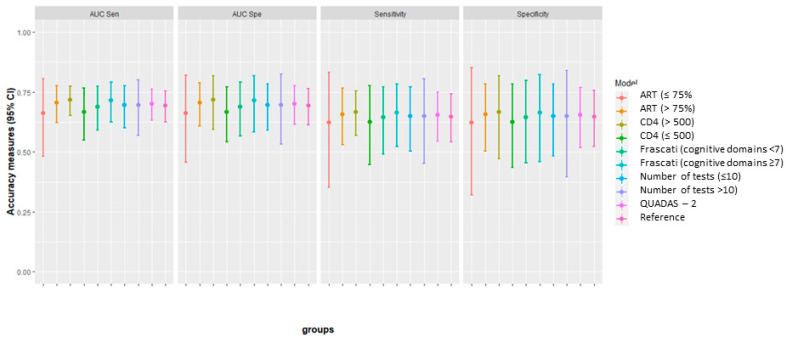
Comparison of sensitivity and specificity with 95% CI between subgroups, at the optimal cut-off point.

An additional summary graph of all the estimation is presented in Figure 7, where we can subjectively appreciate the difference (with the actual studies samples) between the subgroups with regards to the optimal threshold.

## 4. Discussion

The present meta-analysis allowed us to make several key observations.

Although the IHDS seemed to be a good screening test for people living with HIV, based on the available evidence, we found that the scale is not the best discriminating screening test for this population. Nonetheless, the original cut-off score of 10 was optimal for detecting cognitive impairment. For HAND, it offers a sensitivity of 0.646 and a specificity of 0.647. If the testing is intended to evaluate the presence of symptomatic HAND, the use of a threshold of 10 will have a sensitivity of 0.612 and a specificity of 0.742. For detecting HAD, the test will provide a slightly improved sensitivity (0.749) and a specificity of 0.654.

If higher sensitivity is needed, a higher threshold can be used, but this will increase the number of individuals referred for formal cognitive testing and further evaluation. On the other hand, a higher specificity reduces unnecessary referrals, but many true cases could be missed. The present review endorses the use of the cut-off score of 10, which provides a better balance between true positives and false-positive results and could be used to identify individuals that should be repeatedly monitored [67].

Our findings are in line with previous systematic reviews that reported a sensitivity of 0.62 for HAND and 0.74 for HAD [38,39]. However, the previous reviews included different reference standards (e.g., Frascati criteria, American Academy of Neurology AAN, and Memorial Sloan–Kettering MSK criteria).

A possible explanation for the low psychometric properties could be that IHDS evaluates only motor speed, memory, and psychomotor functioning. These domains are frequently affected in HIV patients [13], but older patients may present multiple comorbidities (e.g., Alzheimer′s disease, cardiovascular risk factors, and cerebrovascular disease) [68,69,70]. Therefore, they may also present impairments in other cognitive domains, such as language or visuospatial skills.

The subgroup analysis did not find important differences between different subgroups, including the number of cognitive domains assessed, the number of tests that comprised the reference standard, the level of CD4, or the use of ART. This finding was interesting, as recent studies of neuropsychological batteries used for the diagnosis of HAND found that between 15% and 22% of individuals from an HIV-uninfected control group and 20% of a simulated normal population will score below the threshold for HAND, with false-positive results [18,71].

These errors are generated by two standard practices aimed to increase the sensitivity regarding the mild neurocognitive abnormalities. Firstly, extensive test batteries will determine higher false-positive rates than individual tests, as they require multiple comparisons. There is an increased probability of an abnormal score as the number of tests performed per cognitive domain and the number of assessed domains increases (i.e., diagnosing a normal individual as impaired). Furthermore, the high cut-off scores (including the z scores with a threshold of 1 SD) will increase the overlap between critical portions of test-score distributions in individuals with and without cognitive impairment [18,71]. Therefore, an increased sensitivity will necessarily determine a decrease in specificity. Consequently, the false-positive cases will cause bias in the prevalence estimates and limit analytical estimates′ power [71,72]. However, the Frascati criteria are the most widely used in clinical settings and research, and the direct validation of the criteria for ANI and MND rely on neuropsychological assessment. To date, there are no reliable longitudinal clinical–pathological correlation studies, nor a gold standard antemortem biomarker or imaging finding. The results of the present systematic review and meta-analysis confirm the main potential benefit of IHDS as a test promising to decrease the cognitive assessment time and costs. Nonetheless, it probably has limited uses in discriminating between HAND, symptomatic HAND, and HAD.

In general, we do not recommend the use of IHDS in isolation. A possible solution could consist of using combinations of short tests, including the IHDS, that require 10 to 30 min to complete, enhancing sensitivity and specificity, and could be used in settings with limited resources [42,58]. Further studies could investigate the application of multiple brief screening tests with a full neuropsychological battery to optimize a screening tool that can accurately detect HAND. For example, a recent systematic review showed that the MoCA test, when used at a cut-off of 23, provided a specificity of 0.44 and a sensitivity of 0.79 [44]. Furthermore, the MoCA also investigates cognitive domains such as abstraction, language, and visuospatial abilities that could be impaired in older patients. Therefore, combining both scales, IHDS and MoCA, could improve the accuracy of screening.

In addition, researchers should also consider the value of IHDS in a diagnostic workup to obtain relevant outcomes for patients, such as the benefits of earlier diagnostic and the harms of unnecessary testing.

There is no doubt that there are no perfect screening tests. Therefore, clinicians should consider the ethics and costs of a screening test’s limitations. One first step essential to the development and implementation of screening and referral programs is research to assess any potential program′s acceptability and feasibility from the patient, provider, and clinic system perspectives. Further, as screening programs are developed and implemented, they will also have to positively impact clinical care and patient outcomes [42].

Despite the limitations mentioned above of the IHDS, the objective results of the present screening test are still likely to be more reliable than the information provided by patients or self-reports [73,74]. Individuals with abnormal screening results should be investigated further for the underlying causes of cognitive dysfunction, including mood disorders, cognition-impairing effects of ART, thyroid disease, syphilis, and B12 deficiency [44]. These abnormalities should be identified before referring patients for a further full neuropsychological assessment [75]. A stepwise protocol including cognitive screening would be easy to implement in routine clinical practice, guiding clinicians in dealing with this complex problem [75].

The present study has certain limitations. First, the heterogeneity analysis revealed greater heterogeneity for symptomatic HAND and HAD groups than for the HAND group. Furthermore, the number of studies that reported data on IHDS for detecting symptomatic HAND and HAD is limited compared to the number of studies included in the HAND group. This requires particular caution when interpreting the results, especially in the case of symptomatic HAND and HAD. Second, there was significant heterogeneity among the studies regarding demographic differences, language, cultural, and educational background. The variability in cultural and educational experiences may result in significant differences in performance on neuropsychological tests. Normative corrections (i.e., for age and gender, education, and ethnicity) are not readily available for all populations of HIV patients, or they might be based on a restricted set of demographic factors. This can induce a bias when evaluating cognitive impairment [42,70]. In addition, some other factors may introduce biases, including the total central nervous system penetration-effectiveness (CPE) score, polypharmacy, or medication side effects [76]. Nonetheless, heterogeneity is assumed in diagnostic test accuracy studies, and most approaches of estimating the test accuracy data consider these aspects in the analysis.

## 5. Conclusions

Despite the limitations mentioned above, our systematic review and meta-analysis is the first analysis that compares the IHDS to a reference standard based on the latest diagnostic criteria. We present an accurate comparison between the IHDS thresholds and propose using a common cut-off score of 10 for detecting HAND, symptomatic HAND, or HAD. Although the IHDS test appears to be a practical screening tool for HIV-infected patients, our findings indicate that the optimal threshold for IHDS always comes with a sensitivity–specificity trade-off. The preferred cut point depends on whether sensitivity or specificity is more valuable in a given context.

## Figures and Tables

**Table 1:** Characteristics of the included studies.

Study	Setting	No of Tests	No of Patients	Gender	Reference Test	Age (Years)	Education	CD4 Cells/mL	CD4 Nadir Cells/mL	Viral Load	Percent of Patients on ART
Milanini 2018 [52]	East Africa ^1,2^	6	2208	42% males	Frascati (6 cognitive domains)	39.7 (SD10.7)	44% primary or less	437 (SD 235)	-	2.2 log10copies/mL (SD 2.1)	68%
Trunfio 2018 [53]	Italy ^3^	13	257	72.6% males	Frascati (10 cognitive domains)	51 (44–58)	8 (median)	470 (261–722)	152 (52–271)	1.95 log10copies/mL (1.52–4.43)	-
De Almeida 2017 [54]	Brazil ^4^	17	48	50% males	Frascati (7 cognitive domains)	42.5 (SD 9.1)	9.1 (SD 4.3)	372 (193–547)	88 (28–268)	1.7 (1.7–3.1)	78%
Gouse 2017 [55]	South Africa ^5^	10	94	12.8% males	Frascati (6 cognitive domains)	37.5 (SD 7.57)	9.55 (SD 1.72)	507.41 (14–1413)	-	78.4% undetectable	>75% on ART for more than a year
Mauas 2017 [56]	Argentina ^6^	-	45	84.4% males	Frascati (6 cognitive domains)	37.8 (SD 7.37)	15 (SD3.42)	732 (SD315)	237 (SD 152)	429624 copies/mL (SD 802716)	100%
Lopez 2017 [57]	USA ^7^	27	100	80% males	Frascati (8 cognitive domains)	44.95 (SD 7.63)	10.24 (SD 3.36)	479.93 (SD 224.22)	-	65.1% undetectable	-
Joska 2016 [58]	South Africa and USA ^8^	10	156	37.18% males	Frascati (5 cognitive domains)	40	11	460	144	95% suppressed	100%
Marin-Webb 2016 [59]	Germany ^9^	12	90	98.8% males	Frascati (8 cognitive domains)	43 (IQR 35—51)	15	554	274	-	89%
Ku 2014 [60]	Korea ^10^	10	194	93.8% males	Frascati (6 cognitive domains)	45.12 (range 21—72)	12.8 (SD 3.4)	481.4 (SD 236.0)	187 (SD 138)	82% suppressed	98.9%
Chalermchai 2013 [61]	Thailand ^11^	11	75	44% males	Frascati	33.9 (SD 7)	-	-	-	-	0%
De Almeida 2013 [62]	Brazil ^12^	15	52	46% males	Frascati (7 cognitive domains	42.8 (SD 8.8)	9.1 (SD 4.4)	-	<200 (77%)	44.23% suppressed	69.2%
Rodrigues 2013 [63]	Brazil ^13^	10	187	53.47% males	Frascati (5 cognitive domains)	-	-	-	-	-	65.1%
Van den Dries 2013 [64]	Netherlands ^14^	13	69	82.6% males	Frascati (7 cognitive domains)	53 (SD 11)	-	600 (IQR 430–740)	220 (IQR 60–355)	<200 copies/mL (84%)	100%
Baldez 2011 [66]	Brazil ^15^	8	89	56.18% males	Frascati criteria (6 cognitive domains)	39.87 ± 2.45	10.08 ± 0.79	649.48 ± 59.54 (110–1610)	-	-	55.05%
Joska 2011 [65]	South Africa ^16^	14	96	20.8% males	Frascati (6 cognitive domains)	29.75 (SD 3.67)	10.5 (SD 1.77)	218.09 (SD 150.57)	-	-	0%

Note: ART = anti-retroviral therapy; SD = standard deviation; IQR = interquartile range. ^1^ Ryukyu University Hospital, Okinawa; ^2^ Uganda, Kenya, Tanzania (AFRICOS prospective cohort study); ^3^ Out-patient infectious diseases clinic, Amedeo di Savoia Hospital, Torino; ^4^ Hospital de Clinicas UFPR (HC-UFPR), Curitiba, Southern Brazil (group of 60 + HIV patients); ^5^ Primary healthcare clinics in Khayelitsha, Cape Town; ^6^ Helios Salus HIV Clinic, CABA; ^7^ Patients with Spanish as primary language, recruited from AIDS Service Organizations, including University of California Los Angeles (UCLA), Harbor UCLA Medical Center, AIDS Project Los Angeles, Cedar-Sinai Medical Center; ^8^ Cape Town (South Africa), Baltimore (USA); ^9^ Berlin, Germany; ^10^ Severance Hospital and Korea University Guro Hospital, Seoul; ^11^ Bangkok, Thailand; ^12^ Hospital de Clinicas UFPR (HC-UFPR), Curitiba, Southern Brazil; ^13^ Fundação Oswaldo Cruz (FIOCRUZ) Rio de Janeiro (Brazil); ^14^ Outpatient clinics of the Erasmus Medical Center, Rotterdam (Netherlands); ^15^ Clinic of Infectious Diseases at Fiocruz and the Hospital Municipal Desembargador Leal Junior, Itaborai, Rio de Janeiro (Brazil); ^16^ Primary healthcare centers, Cape Town (South Africa).

**Table 2:** Evaluation of IHDS at different thresholds for detecting HAND.

Threshold	Sensitivity (95% CI)	Specificity (95% CI)
12	0.916 (0.874–0.945)	0.235 (0.154–0.342)
11.5	0.872 (0.813–0.914)	0.332 (0.227–0.456)
11	0.808 (0.730–0.868)	0.444 (0.322–0.574)
10.5	0.724 (0.627–0.803)	0.563 (0.433–0.684)
10	0.619 (0.511–0.717)	0.675 (0.552–0.777)
9.5	0.502 (0.393–0.611)	0.770 (0.665–0.849)
9	0.385 (0. 287–0.494)	0.843 (0.761–0.901)
8.5	0.280 (0.200–0.378)	0.897 (0.837–0.936)
8	0.195 (0.134–0.274)	0.933 (0.892–0.959)
7.5	0.130 (0.087–0.190)	0.957 (0.930–0.974)
7	0.085 (0.056–0.128)	0.973 (0.955–0.984)
6.5	0.055 (0.035–0.084)	0.983 (0.971–0.990)
6	0.035 (0.022–0.054)	0.989 (0.982–0.994)

**Table 3:** The accuracy of IHDS for detecting symptomatic HAND.

Threshold	Sensitivity (95% CI)	Specificity (95% CI)
12	0.918 (0.409–0.995)	0.202 (0.041–0.599)
11.5	0.874 (0.444–0.984)	0.315 (0.101–0.654)
11	0.810 (0.459–0.955)	0.456 (0.215–0.719)
10.5	0.724 (0.425–0.903)	0.604 (0.368–0.800)
10	0.618 (0.308–0.855)	0.736 (0.503–0.884)
9.5	0.500 (0.158–0.842)	0.835 (0.595–0.946)
9	0.381 (0.062–0.851)	0.902 (0.657–0.978)
8.5	0.275 (0.021–0.870)	0.944 (0.704–0.992)
8	0.190 (0.007–0.889)	0.968 (0.742–0.997)
7.5	0.126 (0.002–0.908)	0.982 (0.774–0.999)
7	0.082 (0.001–0.925)	0.990 (0.801–1.000)
6.5	0.052 (0.000–0.939)	0.995 (0.826–1.000)
6	0.033 (0.000–0.951)	0.997 (0.847–1.000)

**Table 4:** The accuracy of IHDS for detecting HAD.

Threshold	Sensitivity (95% CI)	Specificity (95% CI)
12	0.991 0.952–0.999	0.139 0.041–0.383
11.5	0.982 0.912–0.997	0.217 0.068–0.513
11	0.963 0.841–0.992	0.321 0.112–0.641
10.5	0.926 0.727–0.983	0.448 0.177–0.753
10	0.856 0.570–0.964	0.581 0.270–0.838
9.5	0.738 0.394–0.924	0.703 0.387–0.899
9	0.573 0.239–0.852	0.802 0.519–0.938
8.5	0.389 0.129–0.732	0.874 0.647–0.963
8	0.233 0.065–0.570	0.922 0.757–0.978
7.5	0.126 0.031–0.395	0.953 0.841–0.987
7	0.064 0.014–0.246	0.972 0.899–0.993
6.5	0.032 0.006–0.141	0.983 0.938–0.996
6	0.015 0.003–0.078	0.990 0.962–0.998

**Table 5:** Comparison of the optimal threshold of IHDS in different subgroups, based on the risk of bias (QUADAS-2) evaluation.

Subgroup	Optimal Threshold	Sensitivity (95% CI)	Specificity (95% CI)	AUC
High risk of bias studies excluded	10.261	0.655 (0.527–0.764)	0.597 (0.470–0.712)	0.665
All available studies	10.217	0.647 (0.541–0.741)	0.647 (0.522–0.755)	0.683

**Table 6:** Comparison of the optimal threshold of IHDS in different subgroups, based on the number of cognitive domains assessed.

Subgroup	Optimal Threshold	Sensitivity (95% CI)	Specificity (95% CI)	AUC
Assessment of <7 cognitive domains	9.882	0.610 (0.439–0.757)	0.620 (0.451–0.764)	0.652
Assessment of ≥7 cognitive domains	10.281	0.671 (0.510–0.799)	0.647 (0.483–0.783)	0.708
All available studies	10.217	0.647 (0.541–0.741)	0.647 (0.522–0.755)	0.683

**Table 7:** Comparison of the optimal threshold of IHDS in different subgroups, based on the number of cognitive tests used as reference standard.

Subgroup	Optimal Threshold	Sensitivity (95% CI)	Specificity (95% CI)	AUC
Assessment with ≤10 cognitive tests	10.327	0.678 (0.539–0.792)	0.633 (0.485–0.760)	0.703
Assessment of 10 cognitive tests	9.911	0.601 (0.380–0.788)	0.624 (0.403–0.803)	0.648
All available studies	10.217	0.647 (0.541–0.741)	0.647 (0.522–0.755)	0.683

**Table 8:** Comparison of the optimal threshold of IHDS in different subgroups, based on the number of CD4 levels.

Subgroup	Optimal Threshold	Sensitivity (95% CI)	Specificity (95% CI)	AUC
CD4 > 500 cells/mm^3^	10.360	0.653 (0.515–0.770)	0.685 (0.541–0.800)	0.720
CD4 < 500 cells/mm^3^	9.989	0.613 (0.430–0.769)	0.615 (0.433–0.770)	0.651
All available studies	10.217	0.647 (0.541–0.741)	0.647 (0.522–0.755)	0.683

**Table 9:** Comparison of the optimal threshold of IHDS in different subgroups, based on the number of patients receiving ART.

Subgroup	Optimal Threshold	Sensitivity (95% CI)	Specificity (95% CI)	AUC
≤75% of patients on ART	9.585	0.566 (0.291–0.805)	0.625 (0.345–0.840)	0.626
>75% of patients on ART	10.541	0.656 (0.517–0.773)	0.647 (0.512–0.762)	0.698
All available studies	10.217	0.647 (0.541–0.741)	0.647 (0.522–0.755)	0.683

## Data Availability

All data is available within the article.

## Supplementary Materials

The following are available online at https://www.mdpi.com/article/10.3390/diagnostics11061124/s1, S1: Additional data on statistical methods and subgroup analysis: Supplementary Table S1. Goodness of fit for HAND model with successful convergence and non-singularity, Supplementary Table S2. Goodness of fit for HAD models with successful convergence and non-singularity, Supplementary Table S3. Goodness of fit for symptomatic HAND models with successful con-vergence and non-singularity, Supplementary Figure S1. Sensitivity analysis for QUADAS-2: Analysis excluding the studies with high risk of bias, compared with the reference group, Supplementary Figure S2. Sensitivity analysis for studies investigating <7 cognitive domains (according to the Frascati criteria), and ≥7 cognitive domains, Supplementary Figure S3. Sensitivity analysis for studies using ≤10 cognitive tests for the reference standard (accord-ing to the Frascati criteria), >10 cognitive tests, Supplementary Figure S4. Sensitivity analysis for studies with ≤75% of patients on ART and >75% of patients on ART, Supplementary Figure S5. Sensitivity analysis based on the number of CD4.
