# Supplementary material for: International HIV Dementia Scale for HIV-Associated Neurocognitive Disorders: A Systematic Review and Meta-Analysis
Source: Diagnostics (Basel). 2021 Jun 20;11(6):1124. doi: 10.3390/diagnostics11061124 (PMC8235728; doi:10.3390/diagnostics11061124)
Supplement: Supplementary file 1 [file diagnostics-11-01124-s001.zip › diagnostics-1227674-supplementary.pdf]

Supplementary Material

S1. Methods—Model Melection

The selection of the link function (logarithmic or identity) and the distribution (logistic or normal) was made by simple inspection of the data and the fitted model (not presented), where the more plausible choice was the logistic distribution with an identity link for the cutoff. Due to the number of studies per diagnostic subgroup, only a portion of the 16 models was possible to converge and/or without singularity. The convergent and non-singular models for HAND are 12/16 (with data from 13 studies), for HAD are 5/16 (with data from 6 studies), and for symptomatic HAND are 4/16 (with data from 4 studies). From the available models, the ones with the lowest AIC and BIC were selected per group. The best models were:

•HAND: different random intercept (DI) with equal variance (with a $\Delta AIC \geq 2$),

•HAD: constant random Intercept (CI) with unequal variance (with a $\Delta AIC \geq 2$)

•symptomatic HAND constant random slope (CS) with equal variance (with a $\Delta AIC \approx 0$).

Supplementary Table S1. Goodness of fit for HAND model with successful convergence and non-singularity. Equalvar means that an equal variance is assume for sensitivity and specificity.

Models	AIC	BIC
DI Equalvar	238	258
DI	238	262
DICS	240	272
DICS Equalvar	240	270
CIDS	244	276
CIDS Equalvar	244	274
CI Equalvar	260	275
CI	267	284
DS	340	363
DS Equalvar Equalvar	344	365
CS Equalvar	353	367
CS	359	376

Supplementary Table S2. Goodness of fit for HAD models with successful convergence and non-singularity. Equalvar means that an equal variance is assume for sensitivity and specificity.

Models	AIC	BIC
CI	96.6	105
CI Equalvar	98.9	106
DIDS	107.2	128
CS Equalvar	114.4	121
CS	114.5	123

Supplementary Table S3. Goodness of fit for symptomatic HAND models with successful convergence and non-singularity. Equalvar means that an equal variance is assume for sensitivity and specificity.

Models	AIC	BIC
CS Equalvar	40.8	43.2
CI Equalvar	40.9	43.3
CS	41.2	44.1
CI	41.4	44.3

S2. Subgroup Analysis: Covariance Matrix

Covariance Matrices for HAND

$$\begin{bmatrix} 0.066 & 0.051 & -0.001 & -0.001 \\ 0.051 & 0.047 & -0.001 & -0.001 \\ -0.001 & -0.001 & 0 & 0 \\ -0.001 & -0.001 & 0 & 0 \end{bmatrix}$$

Covariance Matrices for HAD

$$\begin{bmatrix} 0.481 & 0.46 & -0.01 & -0.006 \\ 0.46 & 0.913 & -0.006 & -0.105 \\ -0.01 & -0.006 & 0.003 & 0.002 \\ -0.006 & -0.105 & 0.002 & 0.025 \end{bmatrix}$$

Covariance Matrices for symptomatic HAND

$$\begin{bmatrix} 1.73 & 0.479 & -0.576 & -0.106 \\ 0.479 & 4.37 & -0.114 & -1.495 \\ -0.576 & -0.114 & 0.219 & 0.038 \\ -0.106 & -1.495 & 0.038 & 0.55 \end{bmatrix}$$

S3. Sensitivity Analysis

The subgroups analysis was performed using the same model as for HAND, namely different random intercept (DI) with equal variance.

1. The Risk of Bias Assessment (Based on QUADAS-2).

The studies considered at high risk of bias were excluded (Chalermchai 2013, Gouse 2017, Trunfio 2018).

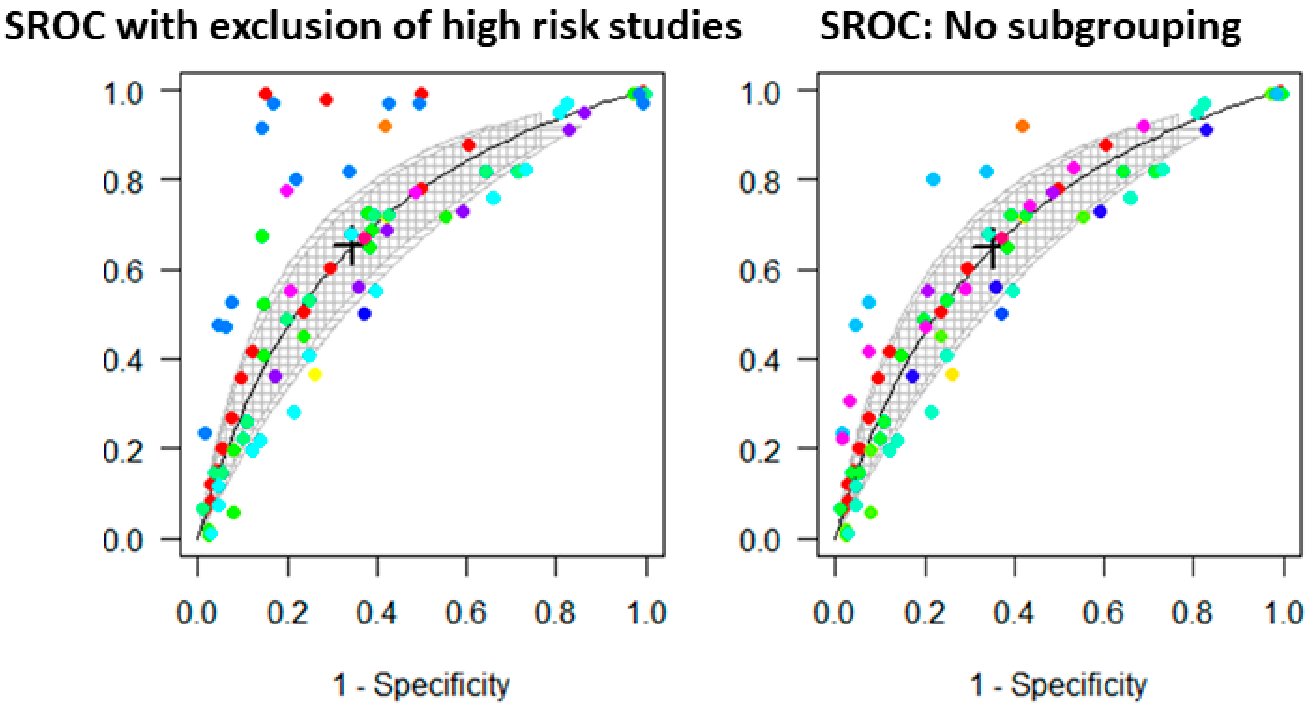

Supplementary Figure S1. Sensitivity analysis for QUADAS-2: Analysis excluding the studies with high risk of bias, compared with the reference group.

2. The Frascati Criteria (<7 Cognitive Domains vs. ≥7 Cognitive Domains)

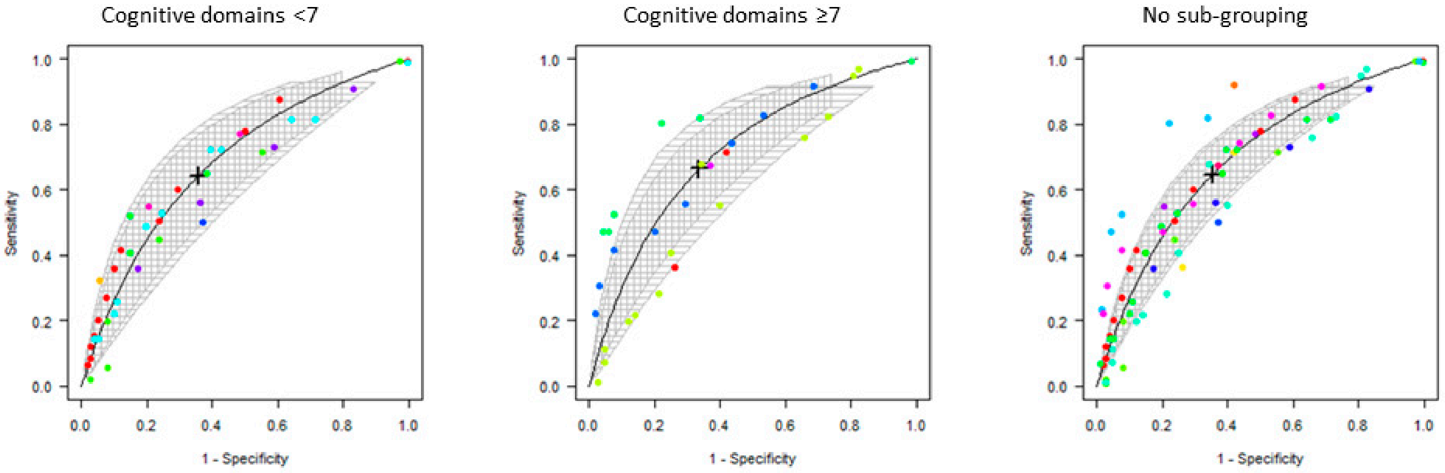

Supplementary Figure S2. Sensitivity analysis for studies investigating < 7 cognitive domains (according to the Frascati criteria), and ≥7 cognitive domains.

3. The number of cognitive tests (≤10 tests vs. >10 tests)

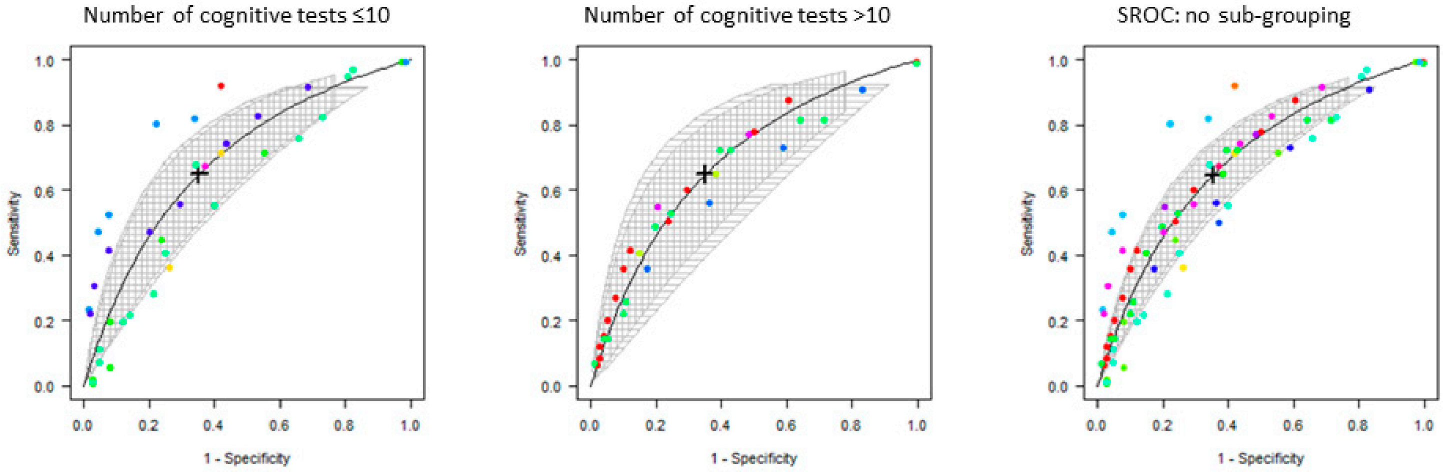

Supplementary Figure S3. Sensitivity analysis for studies using ≤10 cognitive tests for the reference standard (according to the Frascati criteria), >10 cognitive tests.

4. ART therapy (≤75% of patients on ART vs. >75% of patients on ART)

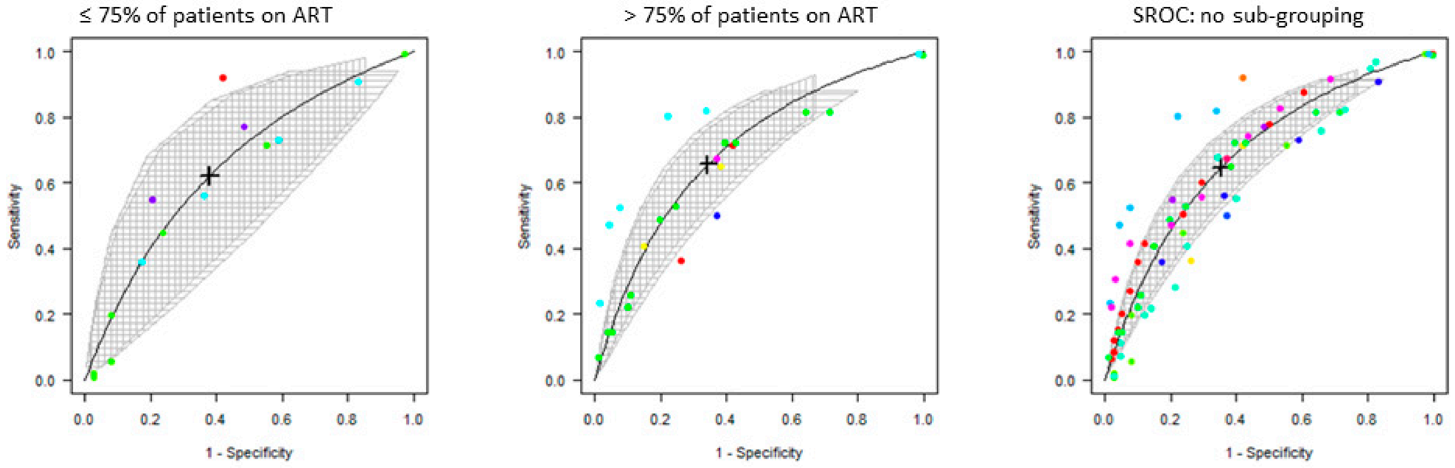

Supplementary Figure S4. Sensitivity analysis for studies with ≤75% of patients on ART and >75% of patients on ART.

5. The number of CD4 (>500 vs. <500)

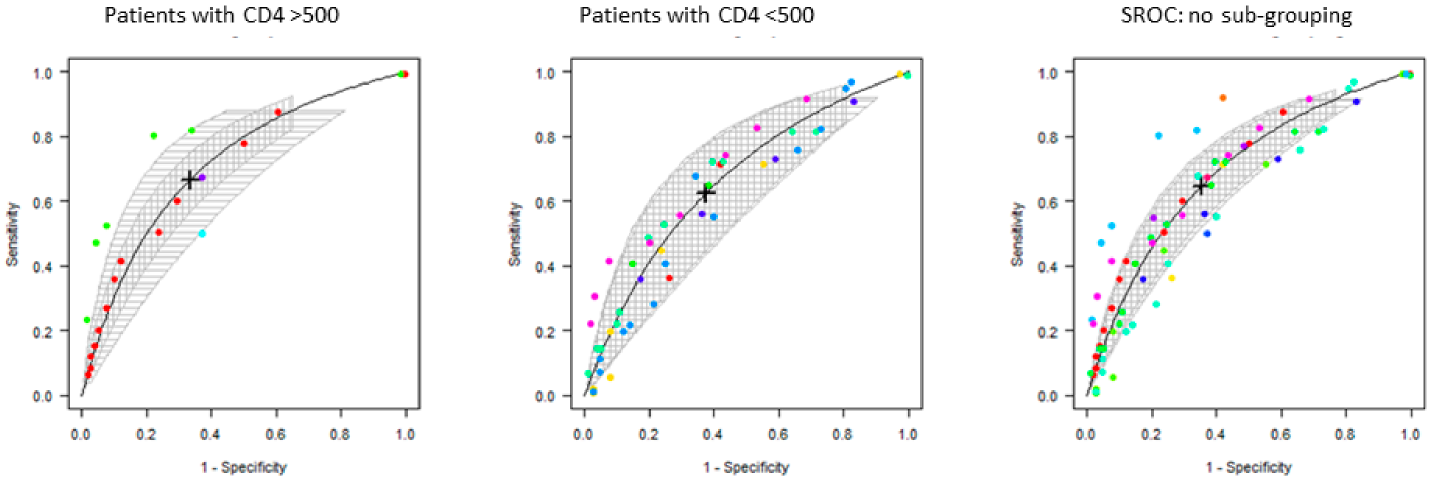

Supplementary Figure S5. Sensitivity analysis based on the number of CD4 (>500 vs. <500).
